# Role of mechanical and thermal damage in pericapsular inflammatory response to injectable silicone in a rabbit model

**DOI:** 10.1371/journal.pone.0216926

**Published:** 2019-05-14

**Authors:** Joon Seok, Soo Hyun Woo, Tae Rin Kwon, Jong Hwan Kim, Guk Jin Jeong, Kapsok Li, Woo Seob Kim, Beom Joon Kim

**Affiliations:** 1 Department of Dermatology, Chung-Ang University College of Medicine, Seoul, Republic of Korea; 2 Graduate School of Medical Science & Engineering, KAIST, Daejeon, Republic of Korea; 3 Department of Plastic and Reconstructive Surgery, Chung-Ang University Graduate School of Medicine, Seoul, Republic of Korea; University Magna Graecia of Catanzaro, ITALY

## Abstract

Silicone is used widely for tissue augmentation in humans. However, late complications, such as delayed inflammation and capsular contracture, remain uncharacterized, despite their importance. In the present study, we aimed to determine whether mechanical and thermal damage induce capsular inflammation around a foreign body, and elucidate the biological mechanism underlying this phenomenon. We injected silicone into the subcutaneous layer of the skin of New Zealand white rabbits. The rabbits were divided into two groups: the control group received no treatment; in the experimental group, external force was applied near the injection silicone using high-intensity focused ultrasound (HIFU). Tissues near the injected silicone were harvested from both groups on Days 4, 7, and 30 after HIFU treatment for comparative analysis. Visual and histological examinations showed clearly increased inflammation in the experimental group compared with that in the control group. Furthermore, capsular tissue from the experimental group displayed markedly increased collagen production. Immunofluorescence revealed marked activation of macrophages in the early stages of inflammation (Days 4 and 7 after HIFU treatment), which decreased on Day 30. Assessment of cytokine activation showed significantly increased expression of heat shock protein (HSP)27, HSP60, HSP70, toll-like receptor (TLR)2, TLR4, and interleukin-8 in the experimental group. The expression of transforming growth factor-β1 did not increase significantly in the experimental group. In conclusion, damage to tissues around the injected silicone induced capsular inflammation. Macrophages and damage-associated molecular pattern molecules were involved in the early stages of inflammation. HSP release activated TLRs, which subsequently activated innate immunity and induced the inflammatory response.

## Introduction

Injectable fillers are used widely in the fields of dermatology and plastic surgery owing to their volume-increasing properties [1, 2]. Although various types of injectable fillers are currently in use (e.g., hyaluronic acid, collagen, poly-l-lactic acid, and polymethylmethacrylate), silicone is reported to be a relatively stable substance within the human body. Therefore, silicone has been widely used as an injectable filler because it is cost-effective, minimally antigenic, and noncarcinogenic [3, 4]. Silicone is commonly used as an injectable filler, for breast augmentation or reconstruction, and as an implant for nasal augmentation. Despite its effectiveness in esthetic and reconstructive medicine, silicone can induce various side effects after injection into the body. These include early complications (e.g., erythema, edema, allergy, and vascular compromise), and late complications (e.g., chronic inflammation, scarring, and foreign body granuloma) [1, 5, 6]. Although most adverse reactions are temporary and relatively mild, late complications associated with inflammation can often result in esthetic or functional damage [7]. When silicone implantation is performed for breast or nasal augmentation, there is a possibility of capsular contracture as a long-term complication. Solidification and deformation of the tissue surrounding the inserted silicone owing to capsular contracture can be painful and esthetically unfavorable. However, the biological mechanism underlying capsular contracture is unclear. One potential cause is inflammation resulting from bacterial contamination, which increases collagen levels and fibroblast proliferation [8, 9]. Similarly, the trigger point and mechanism underlying late inflammation due to injectable fillers remain unknown, although bacterial biofilm creation is considered to be a possible cause [1, 3, 6, 7, 10]. Patients who have been injected with fillers or have received artificial implants are more likely to undergo secondary procedures, including additional filler injection, botulinum toxin injection, filler removal, dental treatment, or additional esthetic procedures using high-intensity focused ultrasound (HIFU). However, the possibility that capsular inflammation is associated with this secondary procedure has not been well explored. As there is a time interval between the silicone injection and the additional procedure, and these are usually performed at different medical clinics, the details of the previous procedure are often unknown. In addition, capsular inflammation does not occur immediately after an additional procedure, but develops over time, so that the possibility of this relationship has often been overlooked. In the present study, we aimed to elucidate the effects of physical damage on the capsular tissue that surrounds the injected silicone. To investigate this, we subcutaneously injected rabbits with polydimethylsiloxane (PDMS), the most commonly used silicone in humans, and treated the rabbits with HIFU. HIFU can be used examine the association between external physical damage and capsular inflammation, as it applies an external force to specific regions surrounding the filler injection site, but does not significantly affect the epidermis or dermis.

## Materials and methods

### Animals and experimental design

The animal experiments were conducted on female New Zealand white rabbits (body weight 2–2.5 kg; Yonam Laboratory Animals, Cheonan, Republic of Korea). During the acclimatization period, general symptoms were observed daily; only healthy animals were used for the test. Anesthesia was induced through the intramuscular injection of ketamine hydrochloride (50 mg/kg of body weight; Ketamine, Yuhan, Korea) and 2% xylazine (0.1 mL/kg; Rompun, Bayer, Germany).

**Fig 1:**
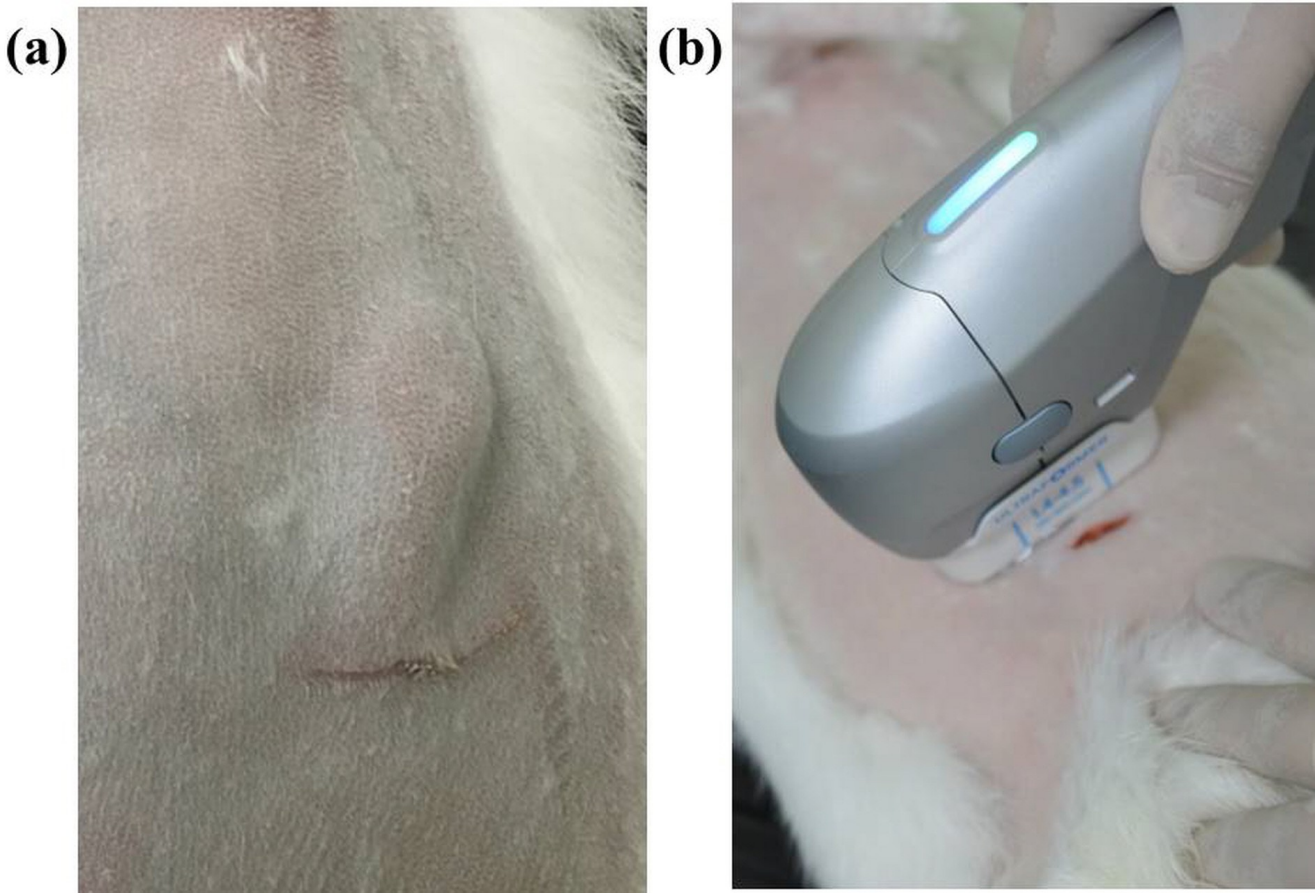
High-intensity focused ultrasound (HIFU) treatment after silicone injection. (a) After making a slit incision on the shaved skin of each rabbit, 1–2 cm^3^ of silicone was injected into the subcutaneous layer. (b) By using HIFU at a depth of 4.5 mm and a power of 1 J, the experimental group was subjected to mechanical and thermal damage at the silicone injection site.

The animals were divided into four experimental groups (in which the silicone was stabilized for 0, 4, 7, and 30 days) comprising two animals each (silicone-only injection and silicone injection followed by HIFU treatment at an energy of 1 J per rabbit (n = 6)). Silicone (approximately 1–2 cm^3^) was injected into the subcutaneous layer and was stabilized by the insertion of a 1 cm^3^ implant (Fig 1A). The rabbits were kept warm during treatment with HIFU throughout the duration of the anesthesia and until they were fully recovered from the anesthetic.

After stabilization for 4, 7, or 30 days, the rabbits were divided into three groups. The rabbits in those three groups were subjected to HIFU treatment by using an ULTRAFORMER III device (Classys Inc., Seoul, Republic of Korea) four times (1 J, 4 MHz, 4.5 mm focal depth) with 2 week intervals between treatments (Fig 1B). Zero, 4, 7, or 30 days after the final HIFU treatment, we sacrificed the experimental animals in a CO_2_ chamber, and analyzed the effect of HIFU irradiation on the silicone.

We determined the location of the HIFU focal point coordinates by heating a 4.5 mm deep silicone gel. All procedures involving animals were conducted in accordance with the guidelines provided by the Care and Use of Laboratory Animals of the National Institutes of Health. The protocol was approved by the Institutional Animal Care and Use Committee of Chung Ang University, Republic of Korea (Protocol Number: 201800016).

### Clinical evaluation

A dermatologist conducted a gross evaluation of the skin surface, dermal damage, and safety. We assessed the clinical symptoms using images captured with a Canon 3000D digital camera (Canon Inc., Tokyo, Japan) at 0, 4, 7, and 30 days after the final HIFU treatment.

### Histological examination

The rabbit skin tissues were fixed with 4% paraformaldehyde (PFA) and embedded in paraffin. Subsequently, we transferred 5-μm sections, cut using a microtome, to ProbeOn Plus slides (Fisher Scientific, Pittsburg, PA, USA), and stained the slides with hematoxylin and eosin (H&E) and Masson’s trichrome. We also stained the sections with Sirius Red (Sigma, Steinheim, Germany) at room temperature for 1 h. After staining, the sections were hydrated with a series of graded concentrations of ethanol, cleared with xylene, and mounted by using neutral resin. All slides were viewed using a Leica DM750 light microscope with an ICC50 HD camera attached (Leica Microsystems Ltd, Switzerland).

### Immunofluorescence analysis

To prepare the sections for immunofluorescence analysis, non-specific binding to the sections was blocked by incubation at room temperature for 2 h with PBS containing 0.2% Triton X-100 and normal horse serum. The sections were incubated at 48°C overnight with a mouse monoclonal antibody against CD68 (1:200, ab955; Abcam, Cambridge, MA, USA). After incubation, the sections were washed three times for 5 min with 0.2% Triton X-100 in PBS, incubated at room temperature with fluorescein isothiocyanate (FITC)-conjugated goat anti-mouse antibody (1:200, sc-2010; Santa Cruz Biotechnology, Santa Cruz, CA, USA) for 30 min. The sections were counterstained with 4′,6-diamidino-2-phenylindole (DAPI) for 5 min. Quantitative data were derived from the immunofluorescent images by pixel analyses using ImagePro image analysis program (Image ProMedia Cybernetics, Rockville, MD).

### Quantitative reverse transcription polymerase chain reaction (qRT-PCR) analysis

Total RNA was extracted from the silicone implanted skin by using an RNeasy Mini Kit (QIAGEN, Hilden, Germany). Complementary DNA (cDNA) synthesis from RNA templates was performed by using a PrimeScript™ RT Master Mix (Takara, Tokyo, Japan). cDNA was obtained and applied to real-time PCR using qPCR 2× PreMIX SYBR Green (Enzynomics, Seoul, Republic of Korea) and a CFX-96 Thermocycler (Bio-Rad, Hercules, CA, USA). The primers used in the reactions are listed in Table 1. The PCR conditions used for the amplification of all genes were: initial denaturation at 95°C for 10 min; followed by 40 cycles of denaturation at 95°C for 15 s, annealing at 60°C for 30 s, and elongation at 72°C for 30 s. The RNA expression data were calculated from the threshold cycle value (Ct) by using the ΔCt quantification method and normalized to the expression of β-actin.

**Table 1:** Primer sequences.

HSP27 forward	5′-CACGAGGAGCGGCAGGACGAG-3′
HSP27 reverse	5′-CAGTGGCGGCAGCAGGGGTGG-3′
HSP60 forward	5′-TGTTTTGGGAGGGGGTTGTGC-3′
HSP60 reverse	5′-AACAGAGAGGCCACACCAGCA-3′
HSP70 forward	5′-CTCCAGCATCCGACAAGAAGC-3′
HSP70 reverse	5′-ACGGTGTTGTGGGGGTTCAGG-3′
IL8 forward	5′-CTCTCTTGGCAACCTTCCTG-3′
IL8 reverse	5′-TTGCACAGTGAGGTCCACTC-3′
TGFβ1 forward	5′-CTTCCGCAAGGACCTGGG-3′
TGFβ1 reverse	5′-CGGGTTGTGCTGGTTGTAC-3′
TLR2 forward	5′-CCGCGGGTTCCCCAGGTTG-3′
TLR2 reverse	5′-GGATCTGGAGCGCCCATCGC-3′
TLR4 forward	5′-GCGGGTGGAGCTGTATCGCC-3′
TLR4 reverse	5′-CTTGGGTTCAGCCGGGCAGG-3′
β-actin forward	5′-GAAATCGTGCGTGACATTAAG-3′
β-actin reverse	5′-CTAGAAGCATTTGCGGTGGACGATGGAGGGGCC-3′

HSP = heat shock protein; IL8 = interleukin-8; TGF = transforming growth factor; TLR = toll-like receptor

### Statistical analysis

The test results were analyzed by using charts grouped by period, group, and are presented as the mean ± standard error. Statistical analysis of the RNA expression data was performed using a paired two-tailed Student’s *t*-test, and significance was accepted for P values of <0.05.

## Results

### Visual examination (gross results)

**Fig 2:**
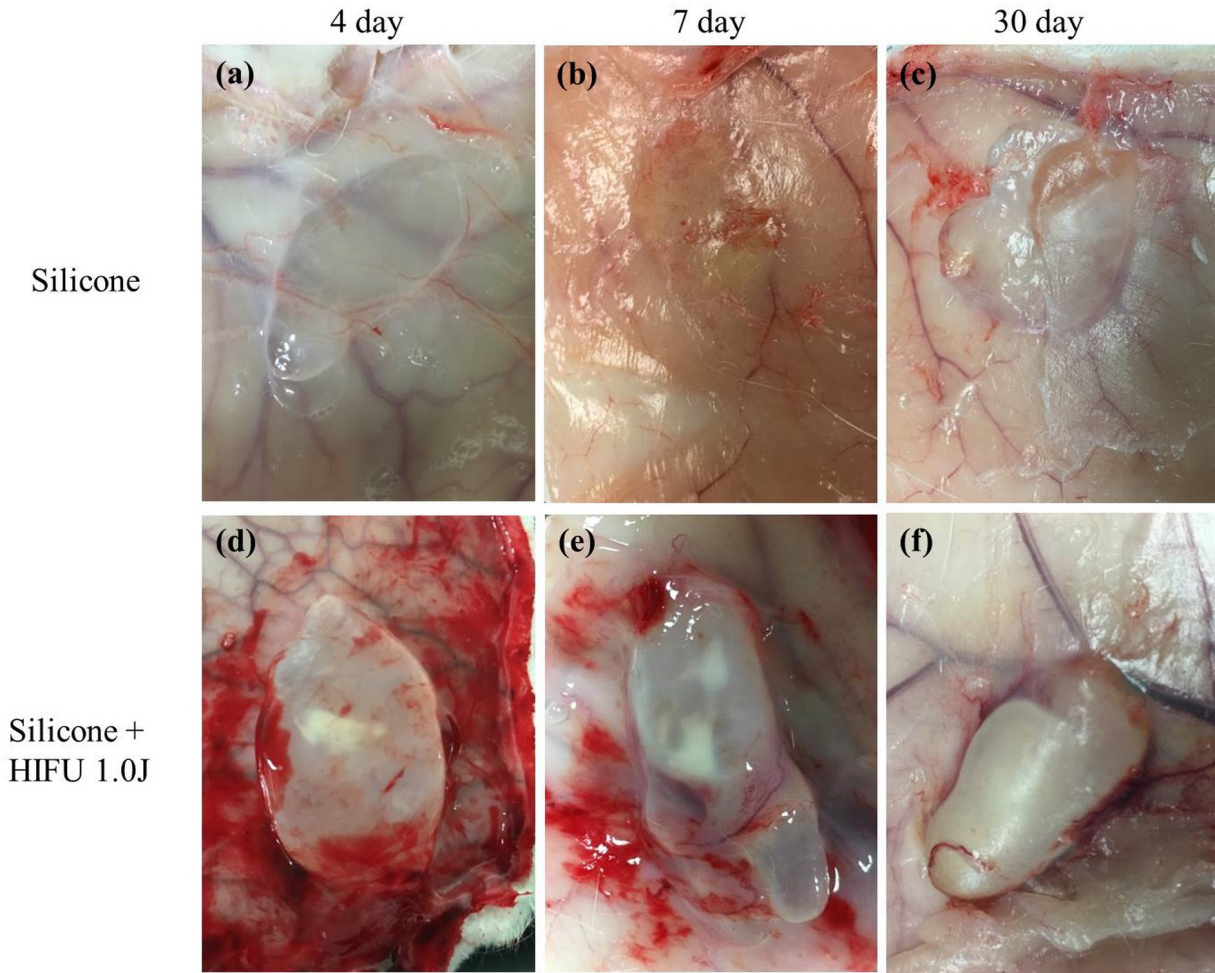
Gross results of high-intensity focused ultrasound (HIFU) treatment after silicone injection. Images collected on Days 4, 7, and 30 after HIFU treatment, to compare tissues from the normal control group (untreated) and the experimental group (treated with HIFU). After each rabbit was euthanized, we evaluated the inner side of the skin (a, b, c). In the control group, the injected silicone was flattened without signs of inflammation, and the capsules were not clearly visible to the naked eye. There was no noticeable difference between the tissues from Days 4, 7, and 30. (d) Visual examination of the tissue from the experimental group on Day 4 after HIFU treatment. Compared with the control group, clear formation of a capsule occurred. (e) Visual examination of the tissue on Day 7. The capsule was more obvious and opaque. (f) Visual examination of the tissue on Day 30. The thickness of the capsule had increased, and progressive fibrosis was obvious. Vascular structure was visible on the outer layer of the capsule. Compared with the normal control group, in which the capsule was flattened, the capsule in the experimental group was bulky and protruding.

In comparison with that in the rabbits in the normal control group, which were not exposed to HIFU treatment after silicone injection (Fig 2A, 2B and 2C), there was a clear formation of a fibrous capsule surrounding the injected silicone on Days 4 (Fig 2D), 7 (Fig 2E), and 30 (Fig 2F) in the rabbits in the experimental group following HIFU treatment. Moreover, the capsules became thicker and opaque over time. Specifically, by Day 30, the capsules were extremely thick and fibrotic based on visual examination, and the vascular structure on the outer layer of each capsule was also visible (Fig 2F). Even visual examination revealed increased inflammation in the capsule due to external damage and the progression of fibrosis and angiogenesis. The injected silicone was flattened in the normal group, but clumped into a ball in the experimental group, possibly owing to capsular contracture, as fibrosis progressed.

### Histology and Sirius red assay results

**Fig 3:**
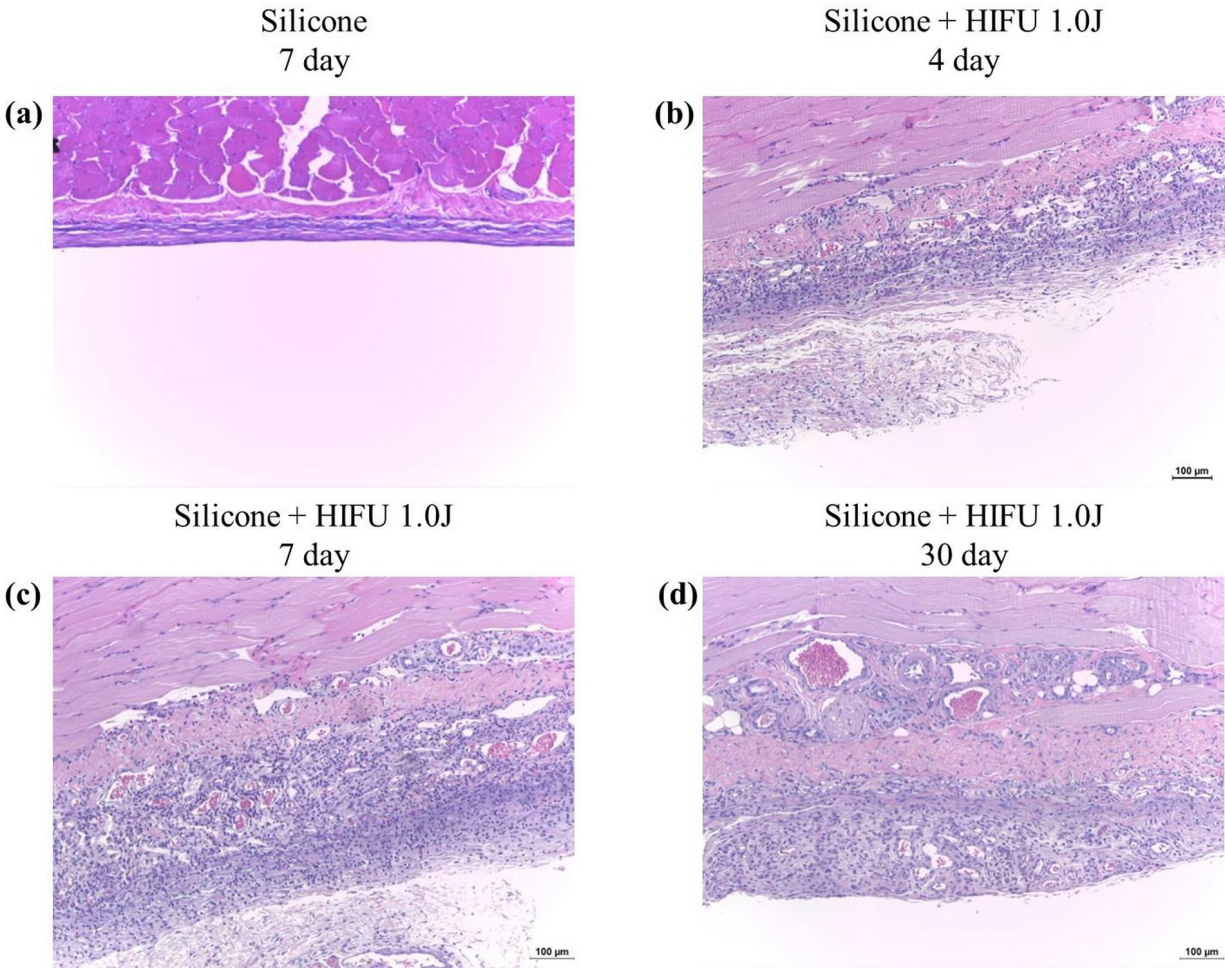
Histologic response to high-intensity focused ultrasound (HIFU) irradiation at silicone injection site. (a) Hematoxylin and eosin (H&E) staining (×100) for the capsule in the control group (silicone injected without HIFU treatment) on Day 7. The capsule was thin and very few inflammatory cells were visible. In contrast, in the experimental group on Days 4 (b), 7 (c), and 30 (d), there were thickened capsules and multiple inflammatory cells surrounding each capsule. There was also obvious angiogenesis in the capsular structure.

The tissues near the injected silicone were harvested from both the control and experimental groups on Days 4, 7, and 30 after HIFU treatment. H&E staining revealed thin capsules and a close-to-zero level of inflammatory cell activity in the control group (Fig 3A). In contrast, the experimental group exhibited thick capsules and active inflammatory cells near the capsule (Fig 3B, 3C and 3D). These findings clearly indicated increased inflammation in the region surrounding the capsule following the application of external force.

**Fig 4:**
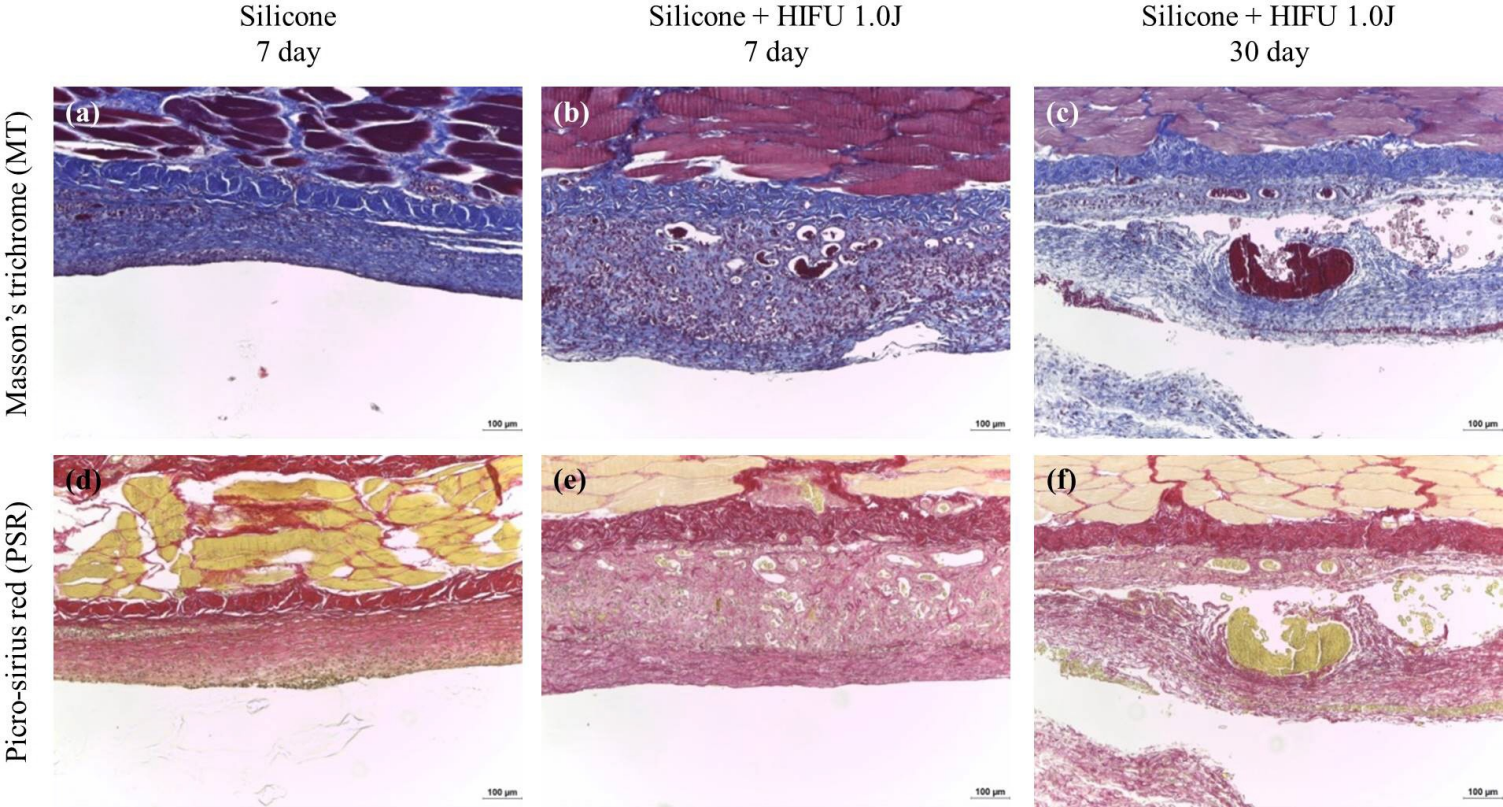
Histologic changes of collagen fibers after high-intensity focused ultrasound (HIFU) irradiation at the silicone injection site. The first row depicts the results of Masson’s trichrome (MT) staining (×100) and the second row depicts the results of Sirius Red staining (×100). Compared with the control group, the experimental group (HIFU treated) had thicker fibrous capsules and more dense, irregular, and disorganized collagen fibers.

The observation of collagen deposition (Fig 4) revealed the thickening of the capsule, and unorganized, dense, and irregular collagen fibers within the capsules in the experimental group compared with the control group. These observations were more evident on Day 30 after HIFU than on Day 7. These observations indicated that collagen deposition and fibrosis worsened over time after the inflammatory response. Furthermore, the experimental group exhibited larger and more extensive vascular structure formation within the capsular tissue (as shown in Figs 3 and 4).

### Immunofluorescence (CD68) results

**Fig 5:**
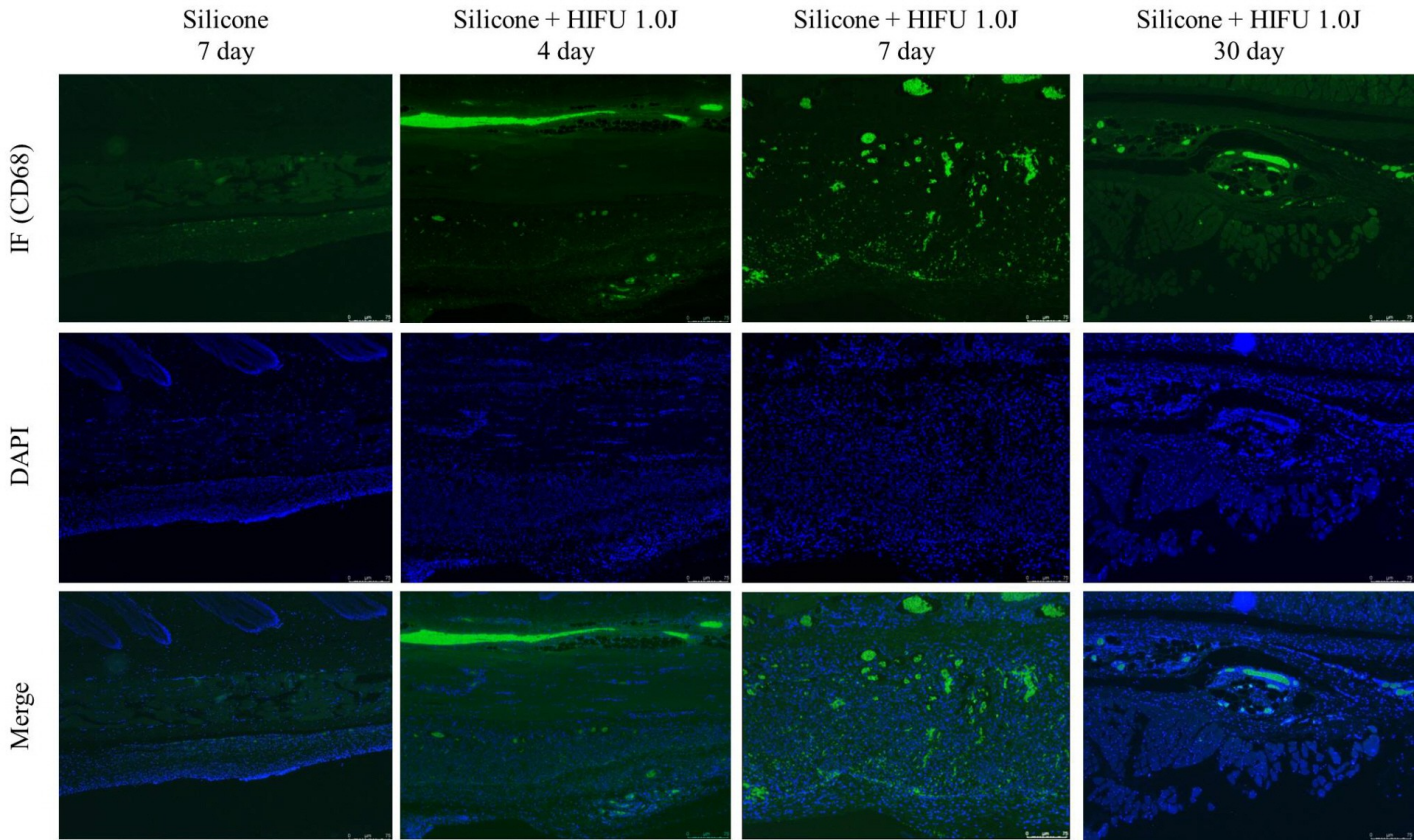
Immunofluorescence (CD68) results of high-intensity focused ultrasound (HIFU) treatment after silicone injection. (a) The first row depicts the immunofluorescence (IF) of the monoclonal antibodies against CD68. The second row depicts the results of staining with 4′,6-diamidino-2-phenylindole (DAPI), and the third row depicts the images of the CD68 and DAPI signals (×100). The first column shows IF staining of CD68 in the control group (untreated) on Day 7. There was minimal activation of macrophages in the control group. The second column shows IF staining in the experimental group (treated with HIFU) on Day 4. Increased IF staining reveals the activation of macrophages. The third column shows the IF staining of the experimental group on Day 7. There was an even greater level of macrophage activation. The fourth column depicts IF staining in the experimental group on Day 30, revealing reduced activity of the macrophages. (b) Expression of CD68 stained by IF (per/field). Markers for the CD68 antigen are most frequently used for the identification of macrophages. (**P < 0.01; ***P < 0.001 vs. the Silicone only group. N = 5).

Although the normal control group showed in negligible immunofluorescence, the experimental group treated with HIFU exhibited increased immunofluorescence on Day 4, and still greater immunofluorescence on Day 7. The signal was subsequently decreased by Day 30. These observations implied that the initial physical damage-induced activation of macrophages, which was greatest in the early stages of inflammation immediately after the application of the external force, then decreased over time (Fig 5A). We quantified the activation of macrophages through pixel analysis, and found statistically significant changes on Day 4 (p<0.001), Day 7 (p<0.001), and Day 30 (p<0.01) (Fig 5B).

### Cytokine activation (mRNA expression)

**Fig 6:**
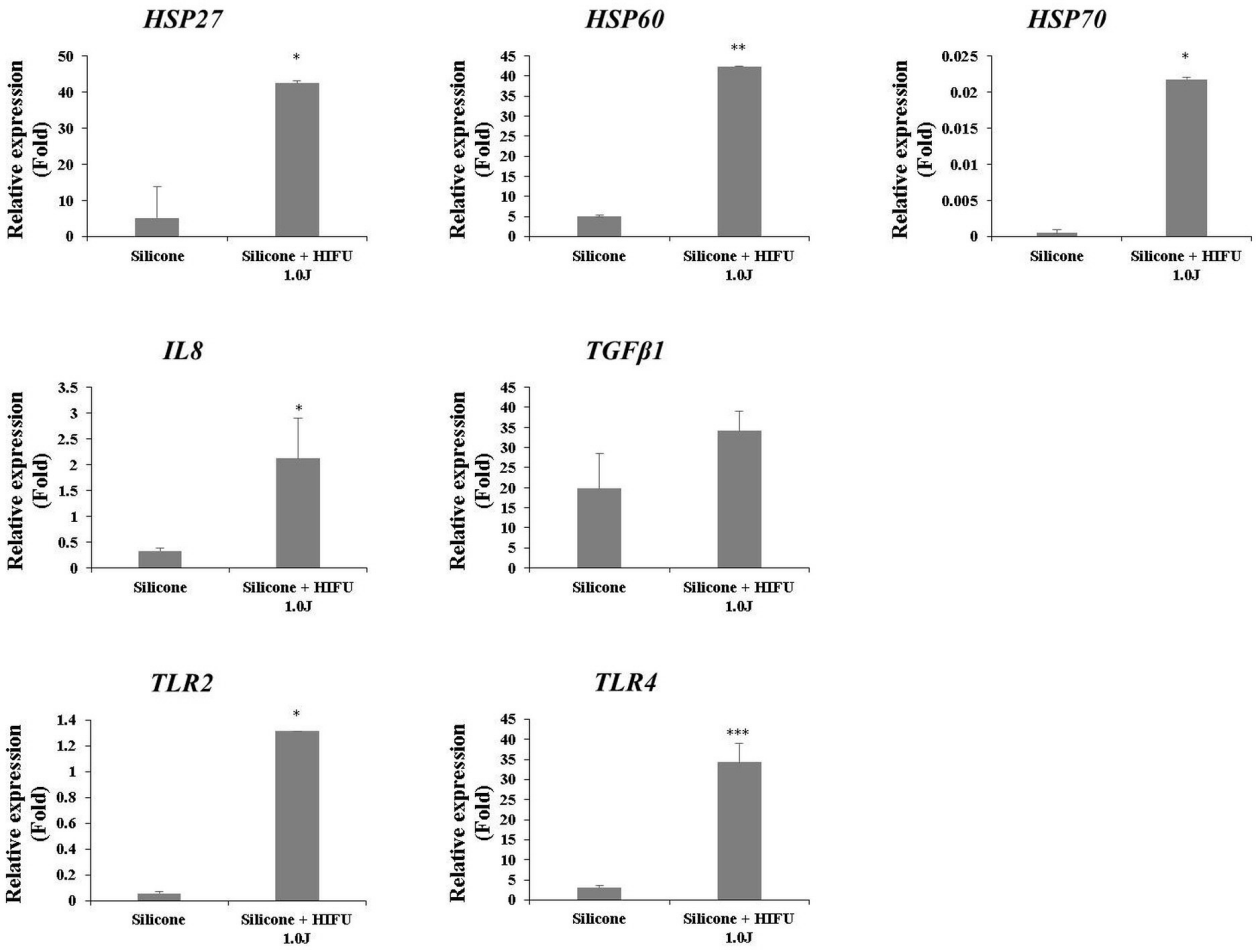
Assessment of cytokine activation (mRNA expression) after high-intensity focused ultrasound (HIFU) irradiation at the silicone injection site. The bar graph depicts the difference in cytokine activation between the control and experimental groups, as revealed by reverse transcription polymerase chain reaction (RT-PCR). The relative expression of heat shock protein (HSP)27, HSP60, HSP70, interleukin (IL)-8, toll-like receptor (TLR)2, and TLR4 were significantly increased in the experimental group that underwent HIFU treatment. The expression of transforming growth factor (TGF)-β1 was increased in the experimental group, which confirmed the trend. However, the difference was not statistically significant. (*P < 0.05, **P < 0.005, ***P < 0.0005 vs the control group).

To quantitatively assess the increase in inflammation and elucidate the underlying mechanism, we harvested tissue on Day 7 after HIFU treatment, and assessed the relative expression of the following proteins by RT-PCR: heat shock protein (HSP)27, HSP60, HSP70, interleukin (IL)-8, transforming growth factor (TGF)-β1, toll-like receptor (TLR)2, and TLR4. TGF-β1 expression was increased in the experimental group, although not significantly. Aside from TGF-β1, the expression of all other cytokines was increased significantly in the experimental group (Fig 6), which suggested a significant increase in capsular inflammation (HSP27: control group, 5.0982 ± 8.5781, experimental group, 42.4198 ± 0.6565, P = 0.0457; HSP60: control group, 5.0982 ± 0.1317, experimental group, 42.4198 ± 0.2663, P = 0.0016; HSP70: control group, 0.0005 ± 0.0004, experimental group, 0.0218 ± 0.0002, P = 0.0165; IL-8: control group, 0.3268 ± 0.0534, experimental group, 2.1189 ± 0.0110, P = 0.014; TGF-β1: control group, 19.7896 ± 8.7695, experimental group, 34.1386 ± 2.3210, P = 0.0856; TLR2: control group, 0.0588 ± 0.0123, experimental group, 1.3149 ± 0.0359, P = 0.0252; TLR4: control group, 3.1456 ± 0.4055, experimental group, 34.3761 ± 0.0373, P = 0.0003).

## Discussion

When foreign material, including both soft tissue fillers and breast or nasal implants, enters the body, it becomes encapsulated via the normal foreign body reaction, regardless of its shape. However, issues can arise when this inflammatory foreign body reaction is overactive [8, 11]. These inflammatory responses are considered critical adverse events, and are difficult to treat [10]. Chronic inflammation near the filler injection site can cause the formation of inflammatory nodules or granulomas, and the inflammatory response to large implants can lead to capsular contracture. Therefore, the prevention of these adverse events is crucial, although the causes and mechanisms underlying these adverse events have not been clearly identified [7, 8, 10].

In the present study, we subcutaneously injected rabbits with PDMS. The injection sites were then subjected to HIFU treatment to demonstrate that the application of an external force can stimulate capsular inflammation at the injection site. HIFU is currently used clinically for skin and subdermal tightening, and no adverse effects have been observed when it is used on normal skin [12–14]; unlike other invasive procedures (e.g., needling), it has minimal effects on the epidermal layer. Consequently, there are no concerns with regard to bleeding or infection from the resulting damage. Furthermore, the depth and intensity of the damage can be controlled, which allows the application of mechanical and thermal damage to a localized region surrounding the foreign body without undesired effects from other factors [12, 13, 15]. In the present study, we performed multiple tests to precisely target the injected silicone using HIFU without affecting the normal tissue, and determined the optimal intensity and depth of HIFU (4 MHz, 4.5 mm). We also applied HIFU to normal skin to confirm that there were no adverse effects, such as the inflammatory response. In a previous study that utilized HIFU for cancer ablation, the absence of tumor antigens during HIFU-based stimulation resulted in a weak immune response and low levels of inflammation [16]. Moreover, the authors of another previous study that utilized HIFU for subcutaneous fat ablation reported that the inflammatory reaction was minimal and subsided over time [15].

The macroscopic results revealed that there was thickening of the capsule, progressive fibrosis, and obvious vessels around the capsule in the experimental group on Day 30, suggesting that angiogenesis had occurred due to inflammation (Fig 2). H&E staining visibly revealed increased activation of inflammatory cells (with mechanical and thermal damage induced by HIFU), thickening of the capsule surrounding the injected silicone, and vascular formation within the capsule (Fig 3) in the experimental group compared with that in the normal control group. These observations implied that external force can act as a trigger for the induction of excessive capsular inflammation. In contrast, normal rabbit skin, which had not been injected with silicone, did not exhibit these outcomes after HIFU treatment.

CD68 immunofluorescence staining revealed increased activation of macrophages in the early stages of inflammation (Days 4 and 7 after HIFU treatment), which decreased over time (Day 30) (Fig 5). Indeed, foreign material entering the body is first recognized by neutrophils, and the foreign bodies that cannot be phagocytosed are attacked by macrophages in the key process of the early inflammatory response [2, 6, 17]. If the macrophages cannot fully remove the foreign body at this stage, multiple macrophages fuse with one another and activate fibroblasts to form a collagenous and fibrous capsule [18]. Although this capsule becomes stable under normal conditions, macrophages with a lifespan of a couple of months may release the phagocytosed foreign body as they undergo apoptosis. Therefore, under normal conditions, inflammation can be reactivated, after a couple of months or even years [6]. In the present study, the application of mechanical and thermal damage resulted in the activation of macrophages in the early stages of the inflammatory response. The application of mechanical and thermal damage to the stabilized capsular structure formed through normal processes induced an inflammatory reaction, and macrophages played a role in the inflammatory response. The results of immunofluorescence staining were quantitated by pixel analysis, which was used to check whether the activation of macrophages was significant at each time point. As the quantity of thermal energy is different for each manufacturer of HIFU equipment, this study used an appropriate standard value of 1 J. It will be possible to obtain the threshold value for HIFU activation of macrophages in subsequent studies. In subsequent studies, comparative experiments will be conducted on the initial energy and instrumental differences. In this study, it is important to note that mechanical and thermal damage can be a factor for the induction of capsular inflammation.

Using tissue harvested on Day 7 after HIFU, when the greatest activity of macrophages was observed, we assessed the expression of various inflammatory cytokines (HSP27, HSP60, HSP70, IL-8, TGF-β1, TLR2, and TLR4) using RT-PCR. Other than TGF-β1, all other inflammatory cytokines exhibited significantly increased expression compared with the experimental group. HSPs are damage-associated molecular pattern (DAMP) molecules that signal endogenous danger during cellular stress or tissue injury [19]. Under normal conditions, HSPs function as chaperones within the cell, resisting cellular apoptosis and providing thermotolerance. However, extracellular HSPs secreted by damaged or stressed cells send signals to nearby cells to induce the stress response and activate the innate immunity of the host [20]. More specifically, HSP27 is well known to activate TLR2 and TLR4, and to induce inflammation [19, 21, 22]. Similarly, HSP70 plays a role in DAMPs and reportedly triggers innate immunity via the TLR4 pathway [23–25]. HSP60 stimulates the host macrophages during processes such as atherosclerosis, and is mostly involved in the TLR4 pathway, although it can also play a role in the TLR2 pathway [20, 26]. Despite the sterile inflammation status in the present study, the increased levels of both TLR2 and TLR4 were probably due to the cellular stress applied to the capsular structure and the subsequent release of HSPs. The excessive inflammatory response, compared with the normal extent of inflammation arising from the reaction to a foreign body, was due to TLRs and the activation of innate immunity caused by DAMP molecules. A previous study revealed that the use of HIFU for cancer ablation enhanced the antitumor immunity of the host owing to the release of HSP70 from the damaged tumor cells [16, 27]. An excessive inflammatory or immune response in the tissue surrounding the foreign material (e.g., silicone) may occur via a similar mechanism.

TLR2 and TLR4 are glycoproteins with key roles in innate immunity. These pattern recognition receptors are expressed on the surfaces of macrophages, neutrophils, and mast cells [19]. The upregulation of TLR2 and TLR4 by HSPs promotes NF-κB transcription within immunocytes, and the secretion of the NF-κB-dependent cytokine, IL-8, and vascular endothelial growth factor [22, 28]. Ultimately, this affects collagen formation and angiogenesis. TGF-β1 and proinflammatory cytokines (e.g., IL-8) are well-known fibrotic factors, and the association between inflammation and fibrosis has been determined [29]. Furthermore, HSPs resulting from heat shock increase the expression of collagen types I and III in human fibroblasts [30]. Although the increase in TGF-β1 expression was not significant, visual examination revealed the thickening and opacification of the capsules, as well as progressive fibrosis (Fig 2). Collagen deposition also increased markedly (Fig 4). These observations implied that these factors had a clear effect on scar tissue formation and fibrosis. The activation of innate immunity by TLRs probably resulted in increased inflammation near the foreign body, the progression of fibrosis, thickening of the capsules, and solidification of the tissue surrounding the foreign body.

Furthermore, the DAMP molecules released by tissue injury or cellular stress can interact with TLRs to produce proinflammatory cytokines, completing the vicious cycle through promoting the secretion of more DAMP molecules [19]. This explain the observation that although the activation of macrophages was increased in the early stages and decreased as time passed, visual examination revealed a continuous deterioration of the inflammatory response over time.

Until now, the causes and trigger points for the formation of granulomas, and the patient cohorts that are susceptible to late complications (e.g., capsular inflammation) at the site of foreign material injection, have been poorly understood [6]. The present study confirms that mechanical and thermal damage from external forces (e.g., HIFU) can activate capsular inflammation, and that the immune response by DAMP molecules (e.g., HSPs) is the biological mechanism underlying this phenomenon. These immune responses were more strongly activated near the silicone (foreign material) compared with the normal tissues.

The present study showed that damage to the filler site or implant site could cause capsular inflammation. Furthermore, fillers and artificial implants other than silicone are expected to result in similar outcomes. Secondary procedures (e.g., HIFU treatment, botulinum toxin injection, or additional filler injection) at the site of previous filler injection should be performed with caution, because they may damage the site. Furthermore, heat stress, which can lead to the release of HSPs, should be avoided, where possible. Finally, it is interesting that increased levels of DAMP molecules, which are often observed in inflammatory diseases (e.g., rheumatoid arthritis, systemic lupus erythematosus, osteoarthritis, and atherosclerosis) and cancer [19, 28, 31] are also observed during capsular inflammation around foreign materials (e.g., silicone) caused by external forces. Therapies targeting this pathway may be useful for the prevention or treatment of capsular inflammation. However, in the present study, external force was applied soon after the injection of the foreign body. Further studies should be performed to determine the effects of external force applied to the foreign body several months or years later (mimicking delayed complications in clinical settings), allowing stabilization of the capsule.

## Conclusion

Mechanical and thermal damage to the tissue near the silicone injection site can activate an inflammatory response through effects on the capsule that surrounds the silicone within the body. The biological mechanism underlying this inflammatory response involves DAMP molecules. The present study confirmed that mechanical and thermal damage to the site of filler injection or implantation can induce capsular inflammation. Patients who receive esthetic procedures often undergo various secondary procedures. Therefore, secondary procedures at the site of filler injection or near implanted foreign bodies should be performed with caution, to prevent the release of HSPs arising from mechanical or heat shock.
